# Muscle contraction: Sliding filament history, sarcomere dynamics and the two Huxleys

**DOI:** 10.21542/gcsp.2016.11

**Published:** 2016-06-30

**Authors:** John M Squire

**Affiliations:** 1 Muscle Contraction Group, School of Physiology, Pharmacology & Neuroscience, Faculty of Biomedical Sciences, University of Bristol, Bristol BS8 1TD, UK; 2 Division of Computational and Systems Medicine, Faculty of Medicine, Imperial College, Exhibition Road, London SW7 1AZ

## Abstract

Despite having all the evidence needed to come to the right conclusions in the middle of the 1800s, it was not until the 1950s that it was realised by two unrelated Huxleys and their collaborators that striated muscle sarcomeres contain overlapping sets of filaments which do not change much in length and which slide past each other when the muscle sarcomere shortens. It then took quite a while to convince others that this was the case, but now the idea of sliding filaments is fundamental to our understanding of how any muscle works. Here a brief overview of the history of the discovery of sliding filaments and the factors that were missed in the 1800s is followed by an analysis of the more recent experiments which have added to the conviction that all muscles operate on the same guiding principles; two sets of sliding filaments, independent force generators and a mechanism of protein rowing that makes the filaments slide.

## Introduction to sarcomere dynamics and sliding filaments

**Figure 1.**
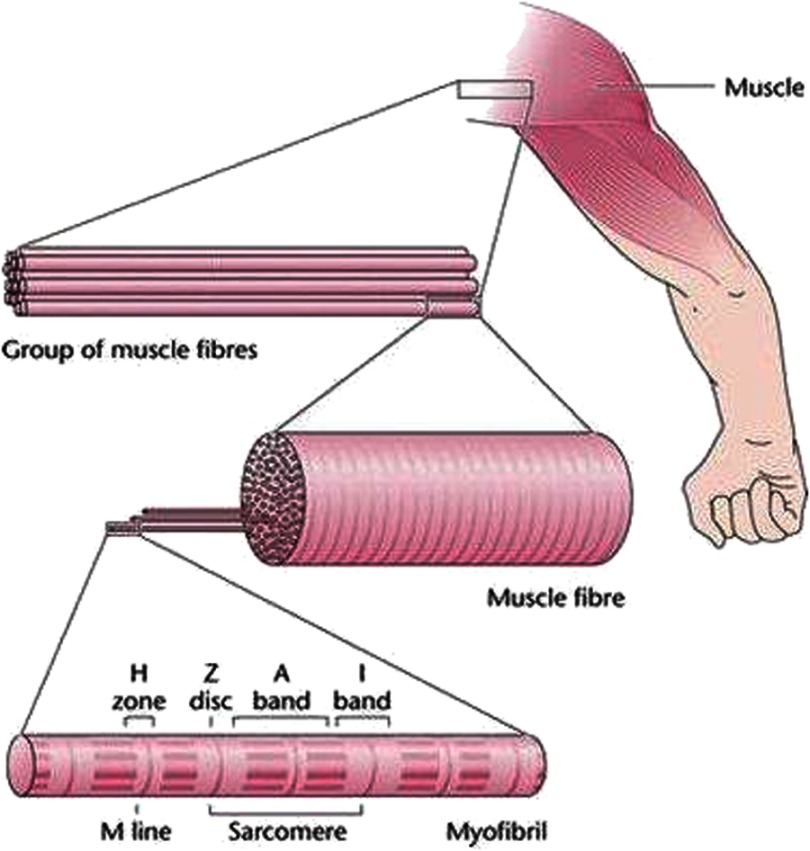
Schematic diagrams showing the hierarchy of structures in vertebrate skeletal muscles, going from an anatomical muscle (top right), to a group of muscle fibres, to a single muscle fibre showing cross-striations and then a single myofibril with sarcomeres, A-bands, I-bands, H-zones, Z-discs (Z-bands, Z-lines) and M-lines (M-bands). Vertebrate striated muscle sarcomeres are often around 2.2 to 2.3 µm long at rest length. Myofibrils may be 1 to 3 µm in diameter and very long, and individual fibres might often be 30 to 100 µm in diameter. Redrawn from Bloom & Fawcett (1975)[t].

**Figure 2.**
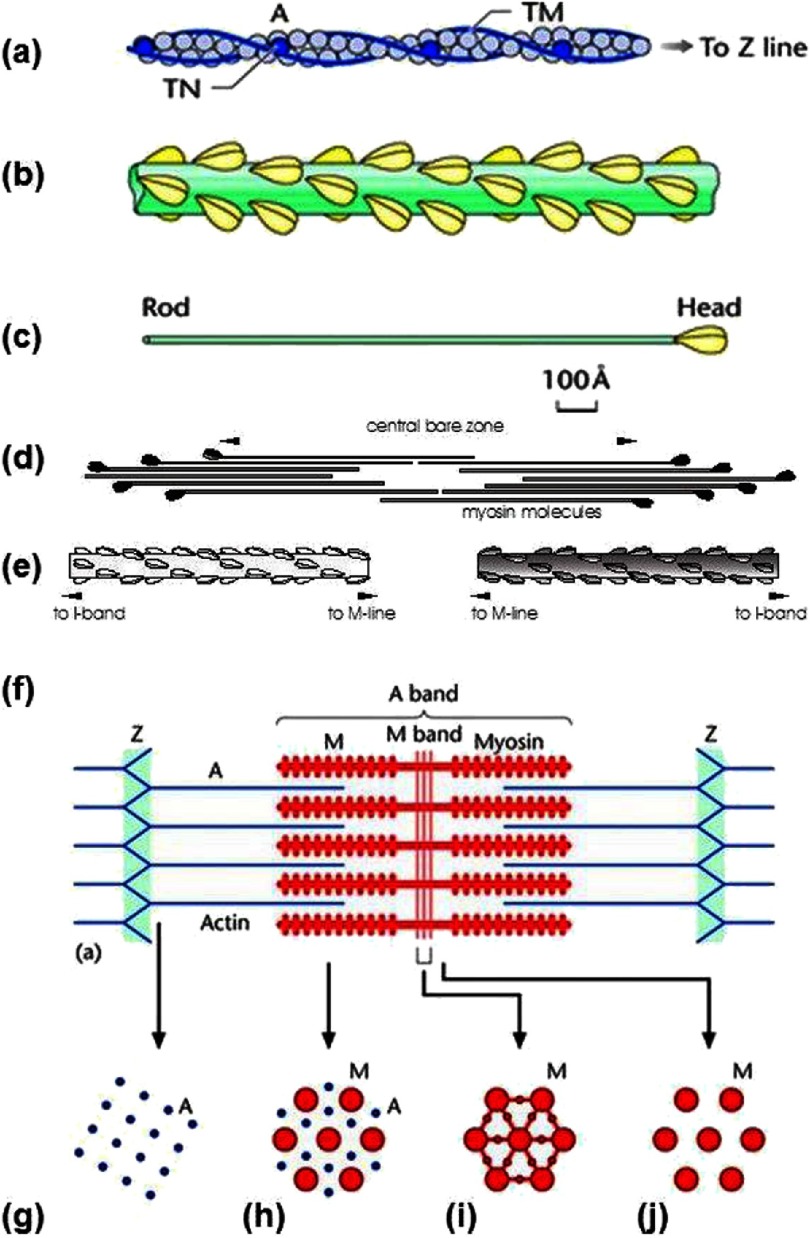
(a) Actin filament composed of actin molecules, A, two tropomyosin strands, TM, and troponin molecule complexes, TN. (b) Bridge region of myosin filament composed of myosin molecules shown in (c) with the rod of the myosin molecules forming the backbone of the filament and the myosin heads arranged on the surface of the filament backbone. (d) The bipolar packing of the myosin molecules showing the anti-parallel arrangement giving rise to a heads-free bare zone region at the centre of the filament. This is also illustrated in (e). (f) Sarcomere structure extending between two successive Z-bands, M: Myosin, A: Actin. (g-j) Cross-sectional views through different parts of the sarcomere, showing (g) the square lattice of actin filaments in the I-band, (h) the hexagonal lattice between overlapping arrays of actin and myosin filaments in the A-band, (i,j) the hexagonal lattice of myosin filaments in the M-band (i) and bare-zone (j) regions, with the extra M- protein density linking the myosin filaments at the M-region in the centre of the sarcomere (i). (From Squire et al., 2005, with permission).

Look at any school biology textbook and the muscle chapter will show a muscle sarcomere, the building block of striated muscles, containing overlapping arrays of myosin and actin filaments (Figures 1 and 2(f)). The idea of muscle filament sliding is now a fundamental concept in biology, but it was not always so. In the 1800s, quite impressive light microscopy of striated muscles showed the sarcomeres to have substructure; a central region (the A-band; although the terminology then was different), which often appeared dark, flanked by two lighter regions (the I-bands) which ended at the Z-discs (or Z-bands or Z-lines). We now know that the A-bands contain filaments of the protein myosin, and the I-bands have filaments of actin, which start at the Z-band, pass through the I-band and overlap the ends of the myosin filaments in the A-band. The part of the A-band not overlapped by actin filaments is called the H-zone (Figures 1 and 2).

Some of the key observations in the studies which outlined the sliding filament model of contraction (Huxley & Hanson, 1954[alp]; Huxley & Niedergerke, 1954[alp]) were actually rather simple (but technically innovative) observations. Firstly, it was found that, as the sarcomere length changed, for example by stretching a relaxed muscle, the length of the A-band remained virtually constant. At the same time the edges of the H-zone appeared to move with the Z-band so that the distance from the Z-band to the H-zone edge remained constant. These two observations alone are almost enough to postulate the presence of sliding filaments, so why were these simple observations missed in the 1800s? [For detailed overviews of the history of muscle research and the conclusions reached see, for example, Needham (1971)[t]; Huxley (1980)[t]; Squire[author] (1981[year]; 1986[year]); McMahon (1984)[t]; Squire & Parry (2005)[t]; Huxley (2008)[t]; Rall (2014)[t].]

## Muscle research in the 1800s

Andrew Huxley, in his fascinating book ‘Reflections on Muscle’ (1980)[t], discusses many ways in which early observations or knowledge of muscle from work in the 1800s was ignored or forgotten by the early 1900s. For example, Boeck (1839)[t] showed that muscle is birefringent, with the slow direction being along the fibre axis. Bowman (1840)[t] knew about fibres, myofibrils, the sarcolemma (muscle fibre membrane) and the presence of striations within each myofibril. Dobie (1849)[t] showed that most of the length change in sarcomeres occurred in the I-band. Brücke (1858)[t] showed that the birefringence is confined to the A-band. He also showed that this birefringence was not increased by stretching the muscle, so it must be due to rodlets which are not themselves stretched when the sarcomere length changes. Kühne (1864)[t] characterised myosin. Krause (1869)[t] showed that the A-band length stays virtually constant when a muscle is stretched and that the high refractive index and birefringence of the A-band were due to birefringent rodlets which extend the whole length of the A-band. He also described how solvents known to remove myosin only extracted material from the A-band, an observation later confirmed by Schipiloff & Danilevsky (1881)[t], so the A-band contains myosin rodlets. When a muscle shortened substantially, dense contraction bands were observed to appear at the Z-band (Engelmann, 1873[p]).

With hindsight we can see that there was probably enough information available by the late 1800s to postulate a sliding filament mechanism for sarcomere shortening, the only really vital piece of missing evidence perhaps being that the I-band (despite being non- birefringent) also contained rodlets. So how did people think muscles contracted in those days? There were a variety of views; Krause believed that the rodlets in consecutive A- bands attracted each other; Engelmann thought that the A-band swelled on muscle activation, mostly in a lateral direction, and that fluid was then drawn into the A-bands. Then, after the observation of transverse and longitudinal elements through muscle fibres (what we now call t-tubules and the sarcoplasmic reticulum) after the introduction of gold chloride staining and in a retrograde step, some authors thought that these structures were rather like the protoplasm of other cells and they tried to suggest that, since all contractile behaviour probably had a common origin, it was the transverse and longitudinal elements which were associated with movement, not the myofibrils. However, Kölliker (1888)[t] argued convincingly that myofibrils are the structures that shorten actively during contraction.

To quote Andrew Huxley from his book ‘Reflections on Muscle’:

“But whatever the rights and wrongs of arguments based on the assumption that all ‘contractility’ is essentially similar, I think there can be no doubt that they helped to reduce the interest that physiologists took in the striation pattern and its changes during contraction”.

So what happened early in the 1900s? One of the first observations was superbly carried out, but rather unfortunate. A new microscope had been developed with the help of the Zeiss works in Jena, Germany. This was an ultraviolet microscope which, with its short wavelength, greatly improved the available resolution. Meigs (1908)[t] used this microscope to study the myofibrils of the ‘asynchronous’ muscles of a fly. The resulting images were beautifully clear and they showed sarcomeres with Z-bands between which there was relatively little substructure. No A-bands or I-bands were apparent, so Miegs concluded that these must be artefacts of the limited resolution of previous microscopes.

What he did not know was that in these particular insect muscles the sarcomere length changes involved in normal contraction are tiny, that the myosin filaments almost fill the whole length of the sarcomere and that the I-bands are very short and not easily seen. Then there was a study by Hürthle (1909)[t] who used cinematography of the leg muscles of the water beetle (*Hydrophilus*), which sometimes showed spontaneous contractions. He followed waves of contraction down these muscles using polarized light and showed that most of the shortening appeared to be in the A-bands; the reverse of the results obtained in the 1800s.

Because he was using photography of living tissue his results were believed and became generally accepted. Other studies in the next few decades appeared to support his conclusions. In addition it was generally believed that myosin was present right through the sarcomere and that the darker appearance of the A-band was due to some other material. How had the field come to such opposite and erroneous views compared with what was known earlier? We should take this as an object lesson in being careful about what we believe.

## Definitive studies in the 1950s

The 1950s were an astonishing time in biology. Following the ravages of the second world war, many scientists, including many refugees from Europe, focused their attention on studies beneficial to mankind, namely on the nature of biological molecules and assemblies. They also had at their fingertips new emerging techniques such as X-ray diffraction and electron microscopy, so the time was ripe for some major discoveries, such as the α-helix structure of protein chains proposed by Pauling et al. (1951)[t].

This was soon followed by the discovery of the DNA double helix by Watson & Crick (1953)[t]. At that time all seemed set fair for significant advances to be made in understanding muscle. But there was an immediate setback. Pauling & Corey (1951)[t], who recognised that the α-helix could be converted to a β-sheet by stretch, thought that this might apply to muscle as well. In this 1951 paper “The structure of hair, muscle and related proteins” they argued that muscles contain continuous filaments through the whole sarcomere and that they can convert from α to β and back as part of the contractile mechanism. However, this conclusion was quickly refuted by Huxley & Perutz (1951)[t]. Perutz had already confirmed the existence of the α-helix by recording the 1.5 Å meridional X-ray reflection, which comes from the axial separation of successive amino acids along a protein chain in an α-helix, using a synthetic polypeptide Poly-γ-benzyl-L-glutamate (Perutz, 1951; see also Squire & Elliott, 1972[p]). In the next paper of the same issue of the journal *Nature*, Perutz and Huxley found that the 1.5 Å peak showed up in X-ray patterns from both stretched and shortened muscle. The α-helices in muscle did not appear to convert to β-structures on stretch. They concluded that: “Our results are incompatible with the mechanism of muscle contraction proposed by Pauling & Corey (1951)[t]....”

So we come to the definitive studies by HE Huxley and Hanson, and AF Huxley and Niedergerke in 1954. Hugh Huxley (1924–2013: Figure 3(b)) studied at Cambridge, United Kingdom, served in the RAF and then started research at the Medical Research Council Unit linked to the Cavendish Laboratory in Cambridge. His early work used X-ray diffraction to study muscle, and also the work with Perutz on the 1.5Å reflection, but his main work used a different sort of X-ray diffraction camera.

**Figure 3.**
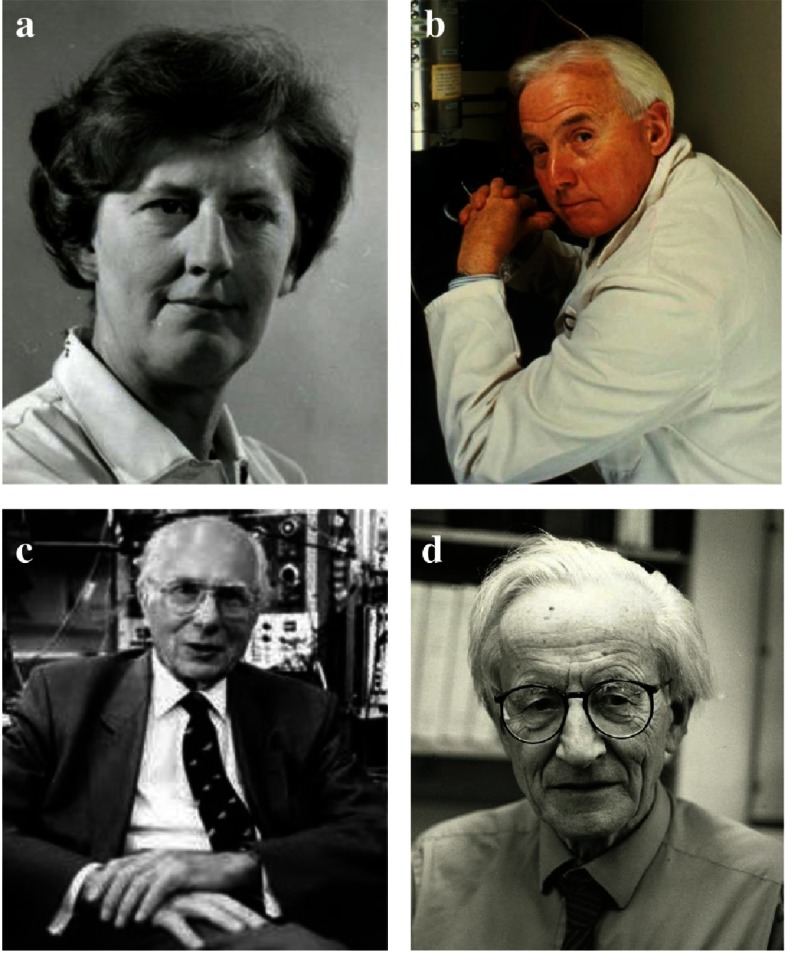
Portraits of the four main protagonists of the sliding filament theory. (a) Jean Hanson, (b) Hugh Huxley, (c) Andrew Huxley, (d) Rolf Niedergerke.

Some of the important axial repeats in myosin and actin filaments are of the order of 350 to 450 Å. The diffraction angles involved in X-ray diffraction are given by Bragg’s Law: *n*λ* = 2d sin *θ**. Here *d* is the spacing involved in the structure doing the diffracting, *n* is any integer, *λ* is the wavelength of the X-rays being diffracted (usually about 1.0 to 1.5 Å in most muscle studies), and the angle of diffraction is *2*θ** (see for example Squire & Knupp, 2005[p]). For a d- spacing as in the α-helix at 1.5Å, sin θ for *n* = 1 is 1.5/2 × 1.5 = 0.5 for a wavelength of 1.5 Å. So 2θ is 60°. If the d spacing involved is 400 Å, then 2θ is only about 0.2° and special X- ray cameras need to be used to study diffraction patterns at such small angles. We’ll discuss later some of Huxley’s results with his low-angle X-ray cameras.

Jean Hanson (1919-1973; Figure 3(a)) was a trained zoologist who in 1948 had joined the Biophysics Research Unit in J.T. Randall’s Department of Physics at King’s College London [See Squire, 2013 for descriptions of this laboratory and of Jean Hanson’s role with Gerald Elliott, who will be mentioned later]. Schick & Hass (1950)[t] and Perry (1951)[t] had shown that preparations of isolated myofibrils could be obtained which showed the normal striation pattern and the normal ATPase activity. Jean Hanson studied these preparations by phase contrast microscopy to see how the striation pattern changed with sarcomere length. Then both she and Hugh Huxley wanted to extend their studies to electron microscopy, which was being successfully employed in Francis Schmitt’s Laboratory at the Massachusetts Institute of Technology (MIT), so they both arrived there independently, Huxley in 1952 and Hanson in 1953. Very soon in 1953 they were working together.

Andrew Huxley (1917-2012; Figure 3(c): later Professor Sir Andrew Huxley PRS, OM, Nobel Laureate; not related to Hugh Huxley) graduated from Trinity College, Cambridge, UK and then in 1939 joined Alan L Hodgkin at the Marine Biology Association at Plymouth and at Cambridge. They worked on and successfully recorded the transmembrane resting and action potentials of the squid giant axon. After the war, Hodgkin and Huxley eventually published their squid axon work in 1945.

Following this, Huxley was joined in Hodgkin’s laboratory at Cambridge by Robert Stämpfli, with whom he published several papers on nerve conduction in frogs. Hodgkin and Huxley then carried out pioneering and definitive experiments on squid giant axons controlled by voltage clamping. This led to five classic papers (Hodgkin et al., 1952; Hodgkin & Huxley, 1952a; 1952b; 1952c; 1952d) and the eventual award of the Nobel Prize in Physiology or Medicine (1963; jointly awarded with JC Eccles).

After his membrane work, and inspired by the work of another giant of the muscle field A.V. Hill, Andrew Huxley started to think about muscle contraction and this was the main focus of the remainder of his long career. Although unrelated to Hugh Huxley, Andrew was part of the famous Huxley family. His grandfather was Thomas Henry Huxley, well-known in the nineteenth century as a supporter of Charles Darwin. Andrew’s half -brothers were the writer Aldous Huxley and the famous biologist Julian Huxley (see Clark, 1968[p]). Interestingly, despite his meteoric career, Andrew Huxley never carried out a PhD; it was not necessary in those days, but he was the only one of our four heroes who was not a doctor. There are interesting comments on PhDs and the British class system from that era in Maruyama (1995)[t] whom we will hear of later in another context.

Andrew had been a near contemporary of David Hill, son of A. V. Hill, at Trinity College Cambridge. They knew each other quite well at Trinity and then, when the second world war intervened, Huxley and David Hill worked together on the application of radar to anti-aircraft gunnery. Interestingly, in the 1914-18 war, A.V. Hill had actually been a pioneer of anti-aircraft gunnery and around 1924 was the main author of the *Text Book of Anti-Aircraft Gunnery*. To quote AF Huxley (1977):

‘This comprehensive two-volume work, issued for H.M Stationery Office in 1924–1925 for the War Office, was still a valuable reference book in the second world war. It was ‘for official use only’ and is not easily found in general libraries …The list of contributors contains at least seven who were, or subsequently became, Fellow of the Royal Society’.

It is also notable that J.T. Randall, who brought Jean Hanson to Kings College (and was also the author’s first boss), was very much involved in developing radar in the second world war. He greatly improved the cavity magnetron, an essential component of centimeter-wavelength radar, which was one of the keys to the Allied victory in the second world war. It is also the key component of microwave ovens.

Rolf Niedergerke (1921-2011; Figure 3(d)), born in Mülheim-Ruhr, West Germany, joined Andrew Huxley’s muscle laboratory in Cambridge in 1952. He had worked on isolated nerve fibres in the Berne Institute of Alexander von Muralt, and was a demonstrator in physiology in Göttingen. He was recommended to Andrew Huxley by Robert Stämpfli as someone who could dissect single intact skeletal muscle fibres, and he also introduced Huxley to many aspects of the available light microscopy techniques.

By 1953 both teams were working on muscle using light microscopy, with Hugh Huxley and Hanson using electron microscopy as well. What did they find and what was different from before?

**Figure 4.**
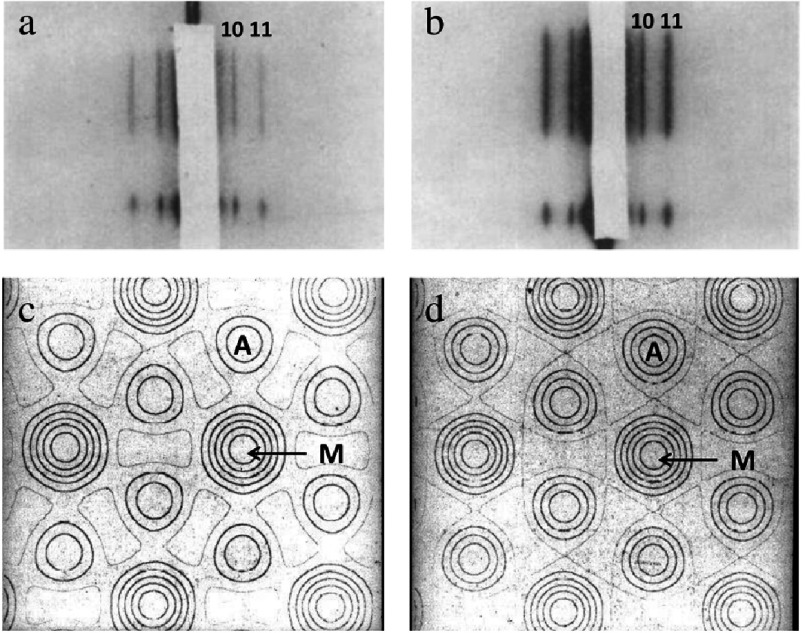
(a, b) Low-angle X-ray diffraction patterns from frog muscle recorded by Hugh Huxley on a 1D or slit camera. The muscle axis was vertical and the diffraction is at right angles to the fibre axis and shows some of the equatorial reflections, labelled 10 and 11. (a) is from resting muscle and (b) from rigor muscle. In (a) the 10 reflection is stronger than the 11; in (b) the 11 reflection is stronger. These observed intensities can be used to generate electron density maps as in (c) and (d), where the myosin filament (M) and actin filament (A) positions can be seen on a hexagonal lattice. In (d) there is much more material at the actin positions than there is in (c), suggesting movement of material (crossbridges, side-pieces) from the myosin filaments towards the actin filaments. Adapted from Huxley (1968)[t].

In that same year Hugh Huxley reported on the X-ray diffraction work that he had done in Cambridge (Huxley, 1953[p]). The first sentence of that paper sets the scene:

“The present day picture of muscle is as follows: muscle is a machine for converting chemical energy into mechanical work; the ‘moving parts’ of this machine are built up of two proteins myosin and actin; the known energy producing reaction most closely linked to the contractile process is the dephosphorylation of adenosine triphosphate (ATP)”. He used low-angle X-ray diffraction, especially of the equator of the diffraction pattern (diffraction at right angles to the fibre axis; Figure 4) to conclude: “the transverse X-ray pattern from living muscle reveals the presence of very long molecules, arranged in a hexagonal array, parallel to the fibre axis and 450 Å apart”.

He goes on to say that when ATP is removed from the muscle, the diffraction pattern changes, but the lateral spacings remain at around 450Å. The axial pattern was also studied and right at the end of that paper Hugh Huxley said: “If the ATP-containing muscle is stretched by up to 40% then the axial pattern remains unchanged. This is rather a surprising result, and it may be an important one. However, there is not time now to discuss its possible implications”.

The first paper produced by Hanson and Hugh Huxley working together at MIT (Hanson & Huxley, 1953[p]) had the ambitious title: “Structural basis of the cross-striations in muscle”. It went quite a long way towards what was needed. Jean’s isolated myofibril preparations were treated with solutions known to extract myosin, and they confirmed that the A-bands in the myofibrils virtually disappeared, leaving only the Z-bands which appeared intact. There was also some ground substance.

The myofibrils were no longer birefringent and would not contract. If the myofibrils were then treated with an actin-extracting solution on the microscope slide, the myofibrils, which in solution virtually collapsed, could be observed to remain structurally intact, but with no A-bands or ground substance. Hanson and Huxley also reported on electron microscopy observations in which they found two sets of filaments in A-band cross-sections, with the second set of filaments also in the I-band and very much thinner than the A-band filaments. The thinner filaments formed a hexagonal ring around a thicker A-band filament, except in the H-zone where the thinner filaments were absent.

Despite this enormous progress, it is clear that they had still not quite grasped what was going on. Their summary was that: “In its simplest form our picture of muscle is as follows: thin filaments of actin extend from the Z-line through the I-band and through one half of the A-band, until they join up with the H-band filaments, the composition of which is unknown. Myosin is located primarily in the A-band, in the form of filaments about 100 Å in diameter, which extend from the A-I junction up to the H-band, where they too join up with the H-band filaments.” So what are these unknown H-band filaments?

## The breakthrough – sliding filaments confirmed

The two classic Nature papers of 1954 started with one by AF Huxley and Rolf Niedergerke. Andrew Huxley was one of those brilliant scientists who could almost do anything, as required. One of his multiplicity of talents was to be able to manufacture his own equipment. As a child he had learnt how to use a lathe, and later in life he used such skills to help with his experiments. He also didn’t waste his time. He is reputed to have thought long and hard about each experiment that he carried out; was it the best way to achieve his aims, would it be reliable, would it answer the right questions? It is said that he spent 90% of his time thinking about the right experiments to do, designing the equipment and so on and then 10% of his time actually doing them. It often then took several years and a great deal of analysis before the results were published, and he had a great analytical mind.

In the case of his early muscle studies, Andrew Huxley was influenced by Niedergerke in his knowledge of microscopy and also his familiarity with some of the papers of the nineteenth century, such as those by Krause (1869)[t] mentioned earlier. Huxley wanted to study intact muscle fibres, which Niedergerke could dissect, but he realised they would be too thick (perhaps 50 to 100 µm) to provide reliable measurements of the sarcomere and A-band lengths and other sarcomere features in the 2–3 µm range using a conventional light or phase contrast microscope. He also realised that an interference microscope could provide what he needed.

Here, the light beam through the microscope is split into two spatially separated beams, one of which goes through the specimen and the other through a background region to serve as a reference beam. The two beams are then recombined and contrast is generated by interference. Such a system can provide an optical section of the specimen. Andrew Huxley made the carcase of his microscope with the optical components being made by Messrs R and J Beck.

[On a personal note, the author’s PhD supervisor, Dr. Arthur Elliott, was another superb scientist who made some of his own equipment, including the toroid X-ray camera which focused X-ray beams using the inside of a hollow toroidal-shaped (i.e. barrel–shaped) mirror 60 to 100 mm long, but only about 3 mm in diameter which Elliott manufactured himself (Elliott, 1965[p]). It was Arthur Elliott who gave Perutz the sample of poly-γ-benzyl-L-glutamate with which Perutz demonstrated the existence of the 1.5 Å reflection from the α-helix (Squire & Vibert, 1987[p]).]

**Figure 5.**
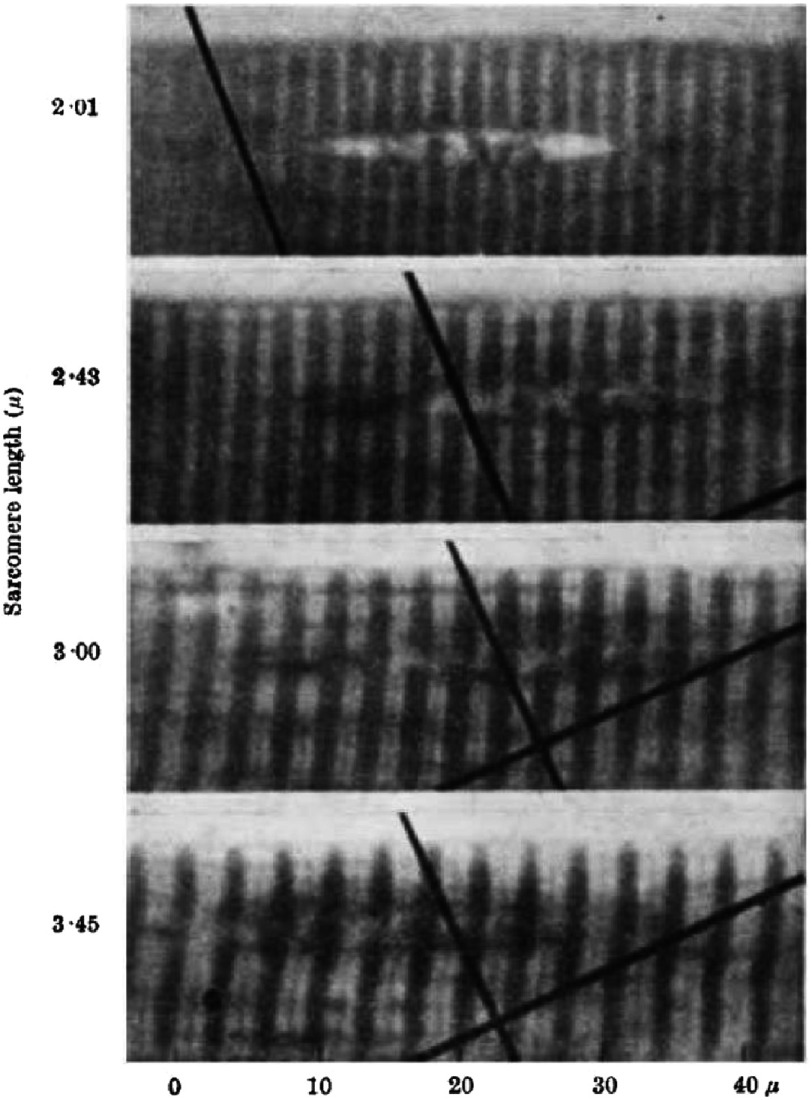
Interference microscopy results of Huxley & Niedergerke (1954)[t] showing what happens when a fibre is passively stretched. Sarcomere lengths are shown on the left hand side. The A-bands (dark) remain almost constant in length as the sarcomere length changes, whereas most of the shortening is in the I-bands (light). Reproduced with permission.

**Figure 6.**
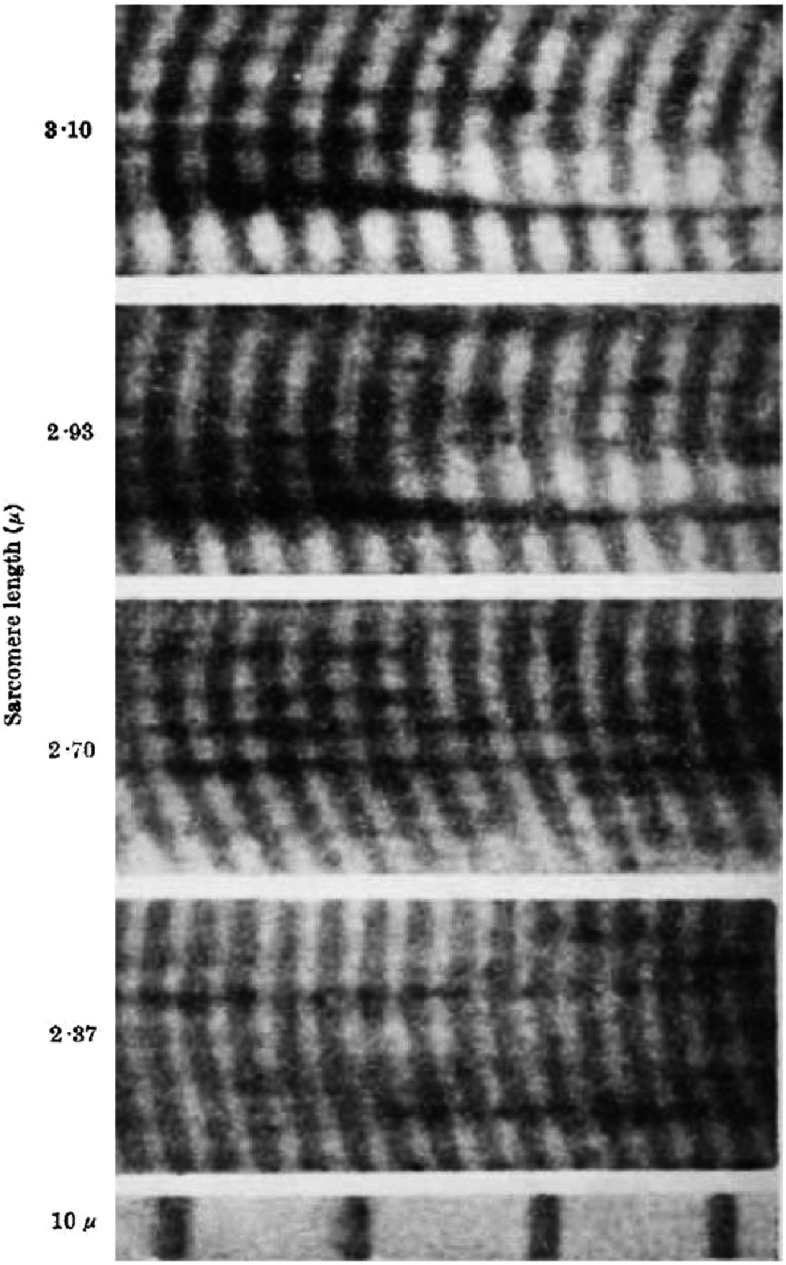
Interference microscopy results of Huxley & Niedergerke (1954)[t] showing what happens when a fibre is stimulated and shortens under constant load. The A- bands stay at almost constant length. Reproduced with permission.

Huxley & Niedergerke (1954)[t] described the results from their interference microscopy of single frog muscle fibres. The contrast in their images could be changed from positive to negative by altering the path difference between the two beams and they found that measurements of the A-band length, for example, were not changed by this procedure. Figures 5 and 6 show some of their results. Figure 5 shows the effects of passive stretch on the fibres, viewed in positive contrast with the A-bands dark. They noted that almost all the change in length within sarcomeres of different length was in the I-band, except at very short sarcomere lengths. They also studied fibres undergoing isometric (constant length) twitches and isotonic (constant load) shortening (Figure 6). In all cases the A-band length was more or less constant except at extreme shortening.

They already knew some of the results from Hugh Huxley on X-ray diffraction from striated muscles (Figure 4), they deduced that there were two sets of filaments, myosin and actin, which did not change length when a muscle shortened unless they ran into an obstruction, such as the Z-band or M-band, and they supposed that if force is generated at a series of points in the region of overlap of the myosin and actin filaments, and each point generated the same force, then the total sarcomeric force should reduce with increasing sarcomere length (reducing overlap) until the actin filaments no longer overlapped the myosin filaments in the A-band. With a measured A-band length of about 1.5 µm and with actin filaments at each end of the sarcomere about 1.0 µm long, then non-overlap should occur at a sarcomere length (S) near to 3.5 µm, a value close to that observed by Ramsey & Street (1940)[t].

Hugh Huxley and Jean Hanson produced the next paper in the same volume of *Nature* as AF Huxley and Niedergerke (Huxley & Hanson, 1954[p]), but reporting on a different approach to the same problem. They were not worried about the sample thickness giving misleading results because they were using Jean’s single myofibril preparations (about 2 µm diameter). They were able to control the myofibril length by serendipitous attachment of the myofibrils to the microscope slide at one end and the coverslip at the other end and also to induce relatively slow contractions by introducing ATP solutions from one side of the coverslip.

**Figure 7.**
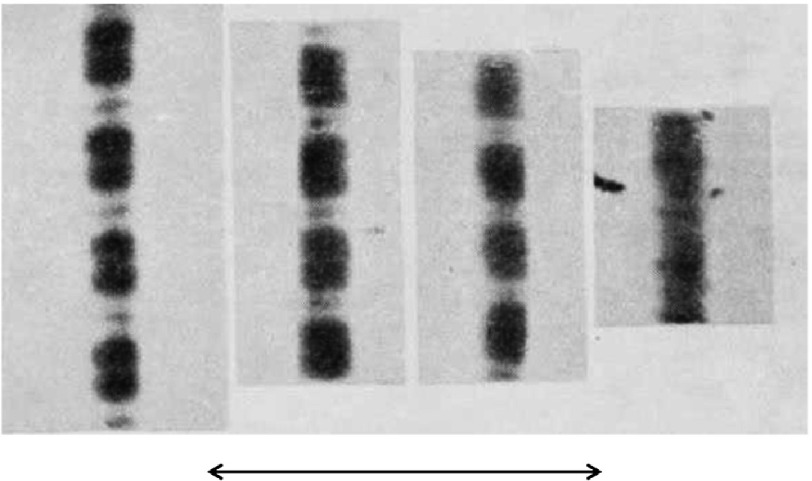
Phase contrast microscopy views of isolated contracting myofibrils as reported by Huxley & Hanson (1954)[t]. The double-headed arrow represents 10 µm. The A-band length remains virtually constant, with the I-band reducing as the sarcomere shortens, and this continues until contraction bands are formed as in the right hand image. Reproduced with permission.

**Figure 8.**
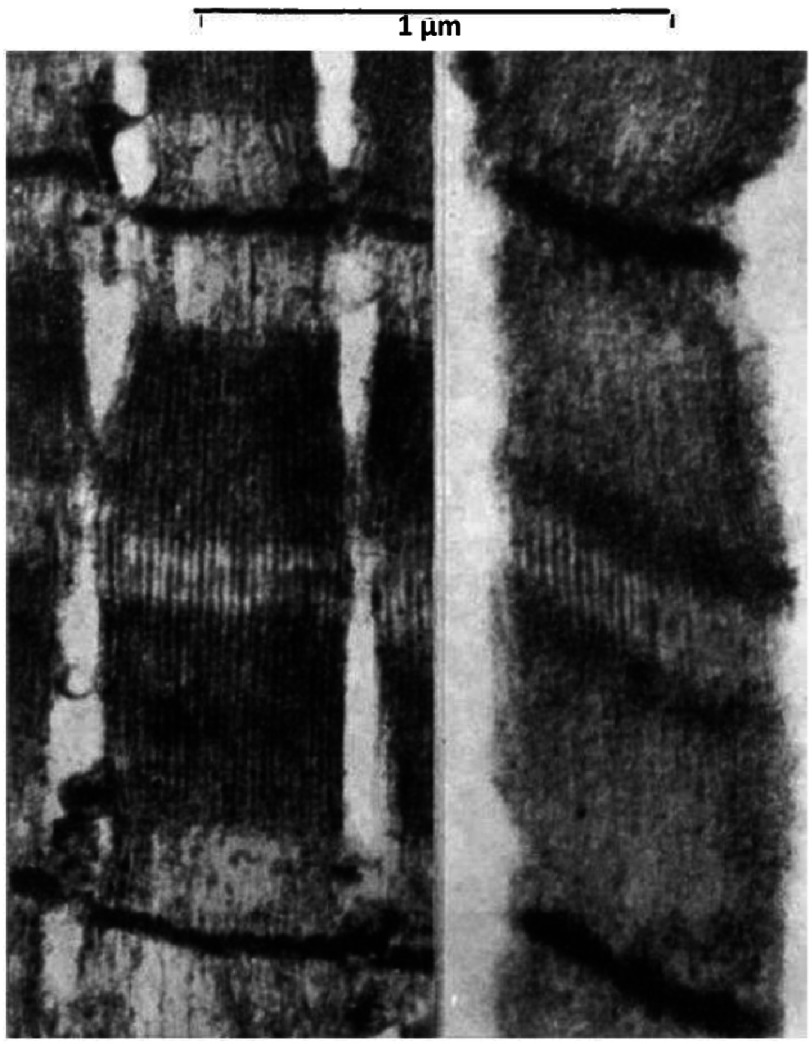
Electron micrographs of rabbit muscle sarcomeres before (a) and after (b) extraction of the A-band material using a Hasselbach & Schneider (1951)[t] solution, taken from Hanson & Huxley (1953)[t]. In (b) most of the A-band has disappeared. Reproduced with permission.

Figure 7 shows the same myofibril induced to contract against zero load from about rest length (∼2.2 µm ) down to about 50% rest length when contraction bands have formed. The left three images show A-bands of almost constant length. [The general features of the sarcomere were also seen in electron micrographs, such as Figure 8 (from their 1953 paper).] The 1954 paper also talked about extraction experiments. Extraction of the A-band material (myosin) left arrays of actin filaments (c) connected to the Z-bands. The myofibrils still showed connectivity (i.e. somehow the Z-bands were linked) so Huxley and Hanson proposed the presence of what they termed S filaments (not visible) across the gap between the ends of the actin filament arrays. Finally they gave their view on how force and movement might be generated. To quote: “A possible driving force for contraction in this model might be the formation of actin–myosin linkages when adenosine triphosphate, having previously displaced actin from myosin, is enzymically split by the myosin. In this way the actin filaments might be drawn into the array of myosin filaments in order to present as many active groups for actomyosin formation as possible.”

The paper goes on to discuss some other ideas, some a bit odd to modern ears, but the idea of cross-connections between myosin and actin were there; structures presumably the same as the ‘points’ discussed by AF Huxley and Niedegerke to explain Ramsey and Street’s length-tension curve dropping to zero at S=3.5 µm.

These two 1954 papers really defined the sliding filament model of muscle contraction, but it was not immediately accepted by everyone in the muscle field. Three later papers, two by Hugh Huxley and Jean Hanson and one by Hugh Huxley alone, all in 1957, helped to establish sliding filaments beyond reasonable doubt.

## Confirmation of sliding filaments

**Figure 9.**
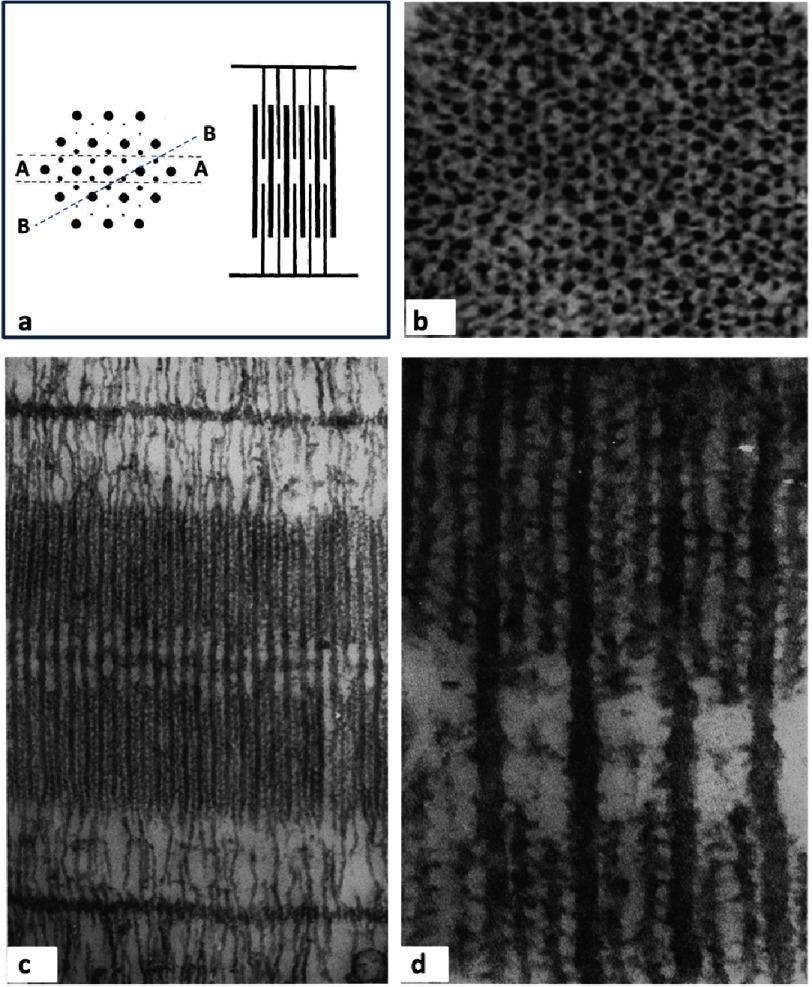
(a) Diagram from Huxley (1957b) showing the hexagonal array of myosin and actin filaments (he called them primary and secondary filaments) and how different longitudinal sections will appear. A section along A-A, and parallel to the fibre axis, which would be as if viewed from the top of the figure, would show alternating myosin and actin filaments, as in the right hand diagram in (a), and as observed in (c). A very thin section along B-B in (a) would show myosin filaments with two actin filaments between them, as observed in the section in (d). Note the clear H-zones in (c, d), where the actin filaments do not penetrate. Reproduced with permission.

Huxley (1957b) included electron micrographs of muscle which have become classics in their own right. Hugh Huxley’s X-ray diffraction studies had shown the presence of filaments in a hexagonal array as in Figures 4 and 9(a). Electron micrographs of transverse sections through the overlap region of the A-band showed exactly the same thing (allowing for some disorder; Figure 9(b)).

Figures 9(a) shows that sections through the lattice and parallel to the filament long axis (longitudinal sections) can show different appearances. Sections cut along the plane A-A, which would be viewed in a direction perpendicular to the dashed lines, would show myosin filaments with two actin filaments on top of each other – they would look like alternating actin and myosin filaments.

Sections cut along the plane B-B, if the sections are thin enough, would show myosin filaments with two separate actin filaments between them. This latter view really does depend on cutting sections that are very thin indeed. Even now this is not at all easy, but this is exactly what Huxley achieved in 1957; Figure 9(c) shows alternating actin and myosin filaments and Figure 9(d) shows myosin filaments with two actin filaments between them.

Figure 9(d) also shows that the actin filaments stop to give the myosin only H-zone in the middle of the A-band. In summary, the actin filaments are not continuous with the myosin filaments, but from the I-band they feed into the gaps between the myosin filaments.

**Figure 10.**
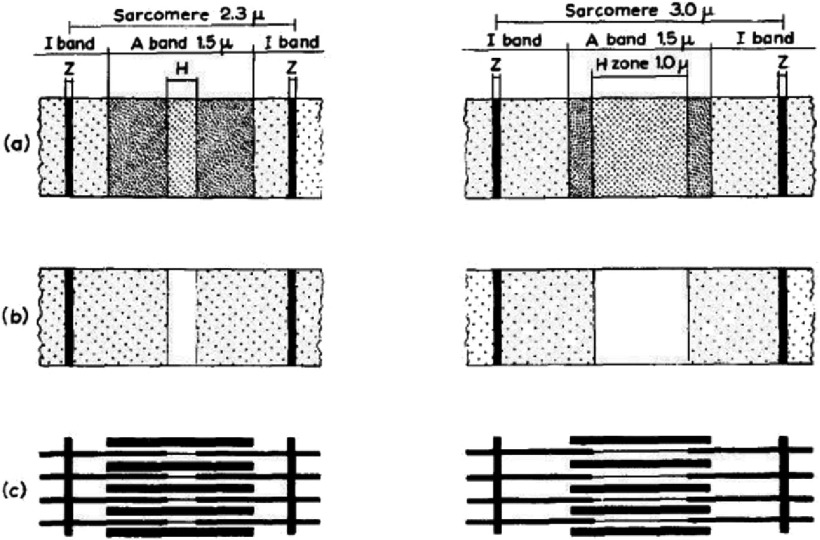
Diagrams taken from Huxley & Hanson (1957)[t] illustrating two myofibrils, one at rest length (2.3 µm, left set of figures) and one stretched to 3.0 µm (right set of figures). (a) The pattern of cross-striations in the intact myofibril, (b) the same after extraction of myosin, and (c) a schematic diagram of the filament including the S-filaments between the ends of the actin filaments. Reproduced with permission.

Huxley & Hanson (1957)[t] used a different approach. They still used myofibrils, but, as in Huxley & Niedergerke (1954)[t], they used interference microscopy because of its clear images, but also because this technique gives information about the relative masses of the different parts of the sarcomere. They showed some of their results diagrammatically as in Figure 10. They showed how the band pattern changed as the sarcomere length changed and how extracting myosin left what appeared to be still well ordered sarcomere ghosts, with just Z-bands and actin filaments somehow connected through what they still termed the S substance (or S filaments).

**Figure 11.**
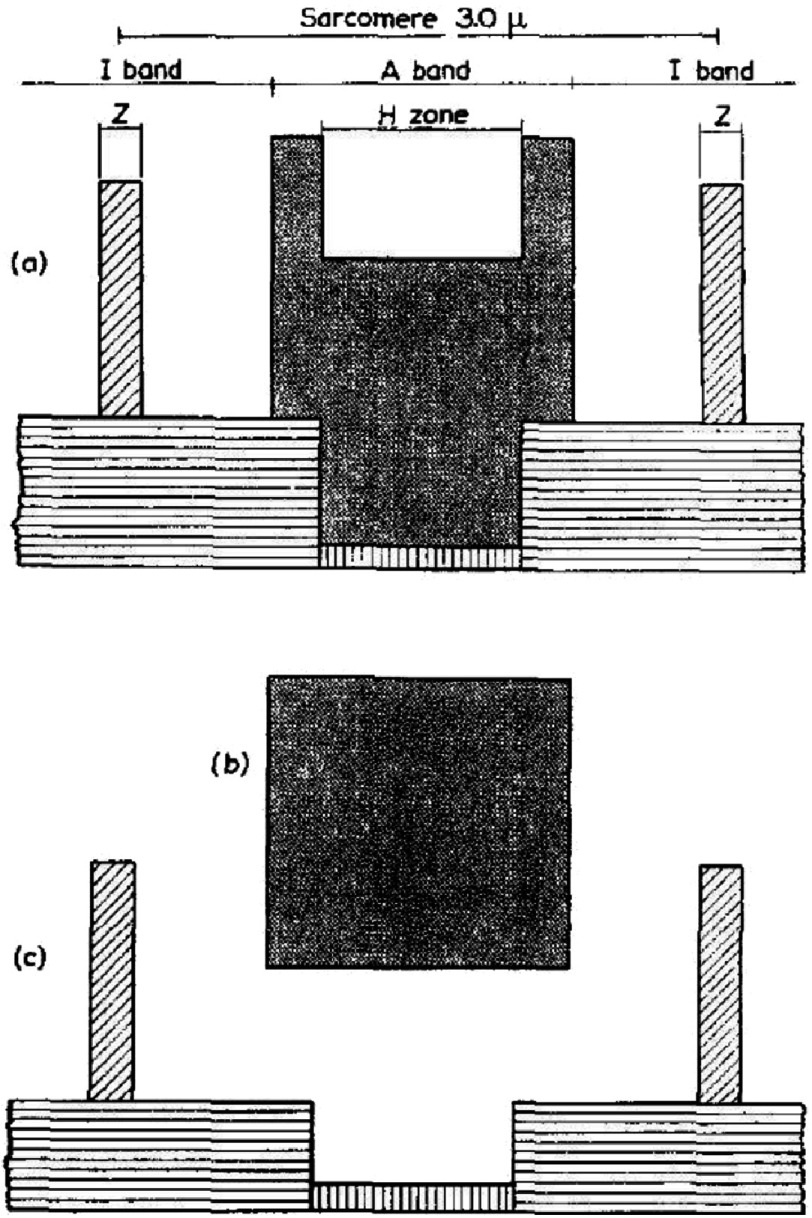
Figure from Huxley & Hanson (1957)[t] showing histograms representing the quantity of protein measured by interference microscopy in different parts of the sarcomere (a) and what happens when the mass of myosin (b) is extracted to leave the mass in (c). Reproduced by permission.

Figure 11 shows the relative densities of various parts of the sarcomere as measured by Huxley and Hanson in their interference microscope. The myosin extracted H-zone (Figure 11(c)) still has a small amount of material due to the S substance. The general conclusions, summarised in Figure 11, were confirmed by Hanson & Huxley (1957)[t] by biochemical quantification of the amounts of myosin, actin and other material in the sarcomere. Results from the different approaches were in good agreement.

I have laboured the point about the S substance because after 1957 it was more or less forgotten about or ignored, just like the nineteenth century ideas about sliding filaments. Later discussions of the sarcomere after 1957 hardly mentioned it. That is until the ground- breaking work of Koscak Maruyama (Maruyama, 1976; Maruyama et al., 1976[p]). He and his colleagues extracted actin and myosin from muscle and found that the residue was an elastic protein that had some similarities to reticulin, but was not the same. He called this new protein connectin.

Then, in one of those unfortunate twists, researchers in Europe rediscovered this protein, found it to be enormous (about 3 mD), the largest protein in existence, and called it titin. This is the name by which it is now universally known, which is a great shame for Maruyama and a great scientific injustice. Those trying to be fair sometimes say titin/ connectin, but usually it is just titin.

**Figure 12.**
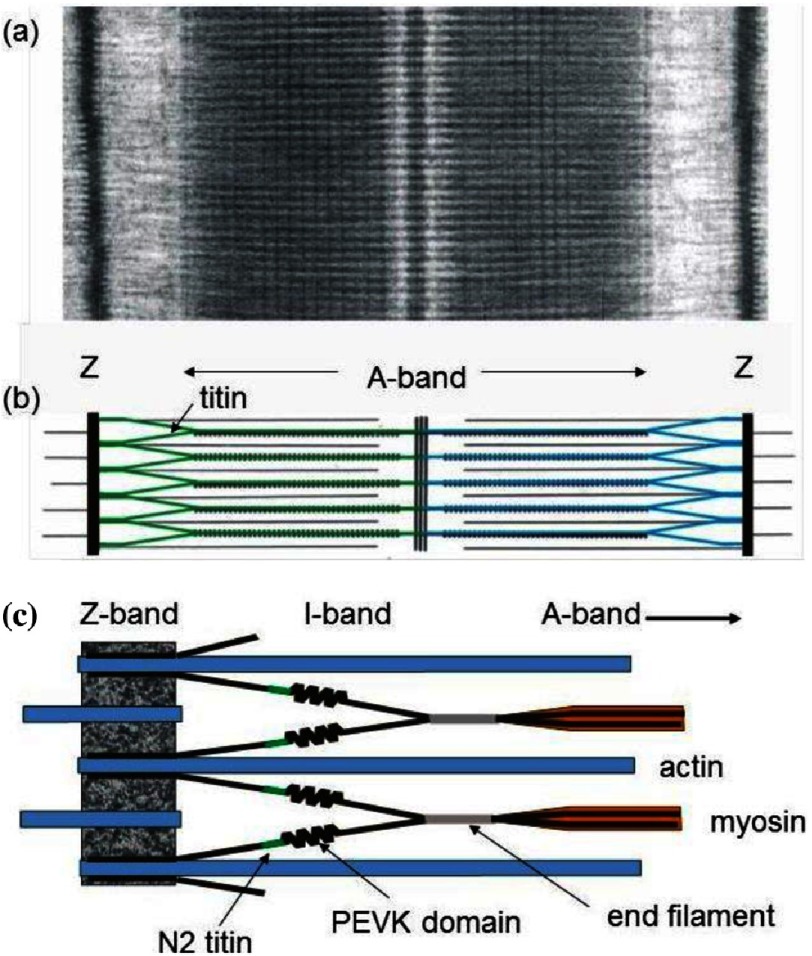
(a) Electron micrograph showing a whole sarcomere from fish muscle in relaxing conditions (Z to Z distance about 2.3 µm). (b) Schematic diagram showing the sarcomere with titin molecules, green and blue, with the N-terminus of each titin molecule located at the Z-band and the C-terminus at the M-band. (c) Magnified version of the area between the Z-band and I-band showing the PEVK and N2 domains of titin and the end filament at the tip of the myosin filament (Trinick, 1981[p]). Adapted from Squire et al. (2005)[t].

Numerous reviews of titin have been published (e.g. Trinick, 1981; Liversage et al., 2001; Knupp et al., 2002; Tskhovrebova & Trinick, 2003; Granzier & Labeit, 2005a; 2005b), so suffice it to say here that it is a very long protein assembled from fibrinectin-like and immunoglobulin-like repeats with some insertions, that it extends from the Z-line through the I-band to connect to the myosin filaments where several titin strands (probably six per half myosin filament) interact with the myosin filament backbone until the bare zone is reached, where titin has a complicated arrangement within the M-band assembly (Figure 12).

Part way along the I-band region is a flexible domain called the PEVK region, which varies considerably in length between muscles and species so that the elastic properties of the sarcomere are matched to the function of the particular muscle and animal. The A-band part of titin has sequence repeats that fit beautifully to the known distribution of myosin along the filaments; so much so that it has been suggested that titin may be involved in defining the vertebrate striated muscle myosin filament length, which we now know to be exactly 1.57 µm (Sjostrom & Squire, 1977[p]).

[On another personal note, the author in the mid-1960s when a humble PhD student in the Kings College Biophysics Department at Drury Lane in London happened to have an office next to that of Jean Hanson, who was by then a Professor (soon to be FRS). The office was shared with Dr. Peter Vibert, another star of the muscle field, who introduced the author to many of the muscle questions of the time. He later became a Church Minister. Hugh Huxley was a very frequent visitor to see Jean Hanson, and they remained friends until Jean’s untimely death in 1973 at the age of only 54 (see Huxley, 2004[p]). Apart from her amazing scientific achievements, Jean will be remembered for her great integrity, for the way that she warmly encouraged newcomers to the field, and for her unfailing kindness.]

## Ideas about swinging crossbridges

While Hugh Huxley and Jean Hanson were doing their structural studies, Andrew Huxley had not been idle. We have seen earlier that sliding filaments were thought to explain the length-tension relationship found by Ramsey & Street (1940)[t], with tension dropping to zero at a sarcomere length (S) of around 3.5 µm. The idea that somehow there might be cross-connections between myosin and actin filaments (sometimes called crossbridges) was first supported by Hugh Huxley’s electron micrographs such as Figure 9(d) which clearly show some transverse densities between filaments and also his X-ray diffraction patterns showing increased mass at the actin filament positions in rigor muscle (no ATP; Figure 4).

It was further supported by the evidence that myosin is the ATPase in muscle and that myosin molecules have a rod portion with globular heads on the end (Figure 2(c); see later study by Lowey et al., 1969[p]).

**Figure 13.**
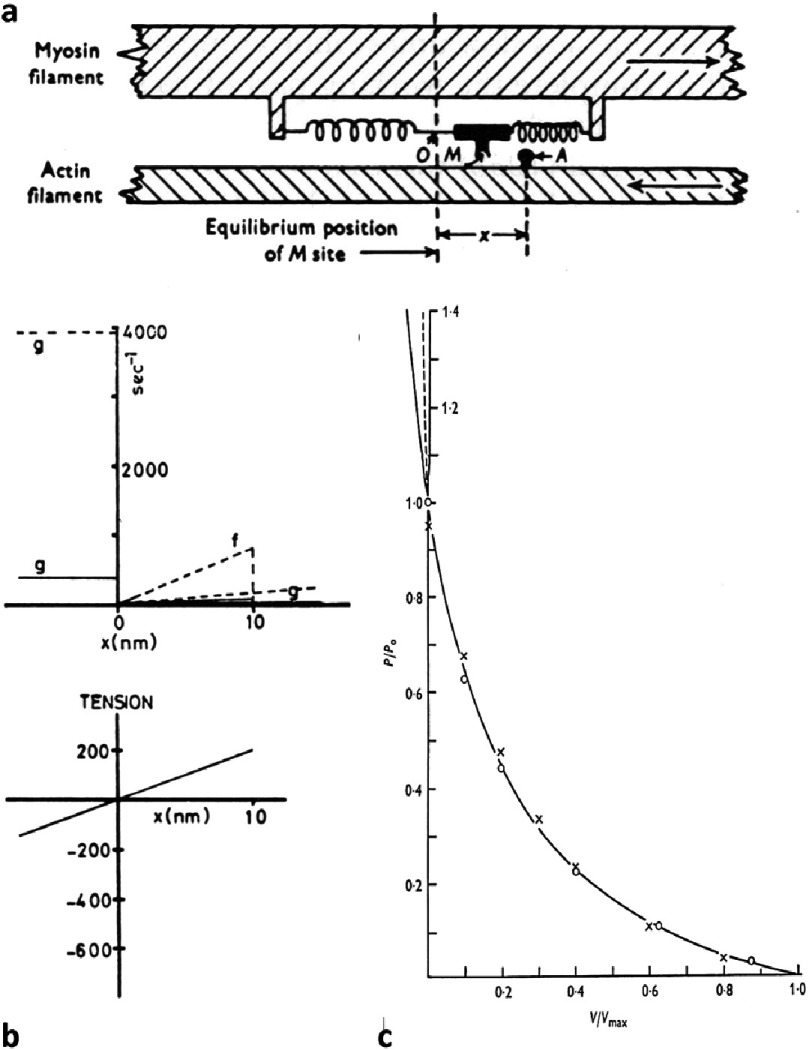
Diagrams from Huxley (1957a) concerning the action of side-pieces (crossbridges) on myosin filaments with an attachment rate to actin of f and a detachment rate of g. (a) shows the basic idea with the definition of the displacement x. The way that f and g vary with x is shown in the top part of (b) and the variation of tension with x is in the lower figure. Huxley’s calculations showed that this model would fit the force velocity curve of AV Hill ((c); Hill, 1938[p]). The solid line is the Hill equation and the circles are values calculated from the Huxley (1957a) model.

On this basis Andrew Huxley produced in 1957 a monumental paper on the theory of a possible myosin crossbridge cycle (note that he called the crossbridges, side-pieces). His model is illustrated in Figure 13(a). He thought about crossbridge kinetics and described a cycle of crossbridge attachment to and detachment from actin governed by two rate constants, *f* for attachment and *g* for detachment. By specifying how *f* and *g* and the tension of attached crossbridges might change (Figure 13(b)) as a function of the relative positions of actin and myosin filaments sliding past each other (the value of x in Figure 13(a)), Andrew Huxley was able to explain some of the key physiological features of muscle behaviour. This included the force velocity curve in Figure 13(c) which had been fitted empirically by A.V. Hill in what is termed the Hill Equation (Hill, 1938[p]).

**Figure 14.**
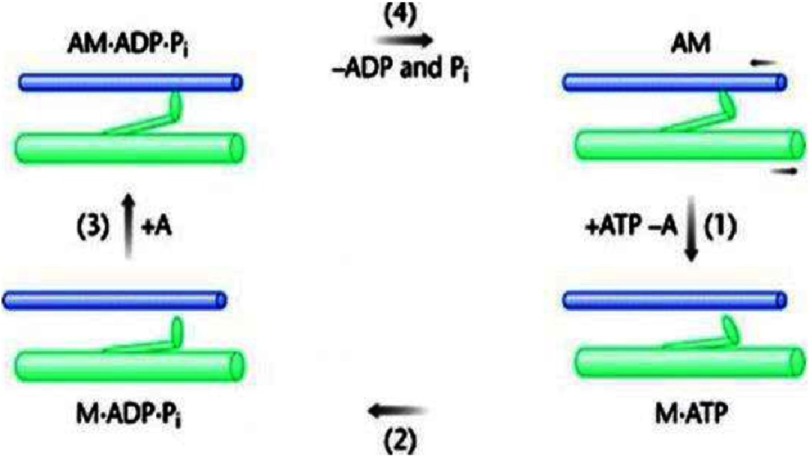
The actomyosin ATPase cycle as determined by Lymn & Taylor (1971)[t], together with the structural crossbridge cycle of Huxley (1969)[t]. In the rigor state (AM; top right) crossbridges (myosin heads) are rigidly attached to actin in a specific conformation at a ‘45°’ angle forming the AM rigor complex. When ATP is added, the crossbridge is released from actin (1) and hydrolysis of ATP into its products, ADP and Pi, occurs, with both products still attached to the crossbridge (2). The hydrolysis of ATP is assumed to be accompanied by a reverse conformational change of the heads back from ‘45°’ to ‘90°’. It is the M.ADP.Pi state that can rebind to actin (step 3) with the crossbridges still at a ‘90°’ angle and forming AM.ADP.Pi. The transition from AM.ADP.Pi to AM.ADP to AM, possibly with some isomerisation steps within each state, is associated with force production and movement. The swinging of the elongated attached crossbridges from ‘90°’ to ‘45°’ will cause relative sliding of the myosin and actin filaments if they are free to move.

Ideas that elongated myosin heads might swing on actin to produce force and movement were boosted by the results of Reedy et al. (1965)[t] on crossbridge appearances in longitudinal sections of insect flight muscle. They found that images from resting muscle appeared to show cross-connections at about 90° to the long axis of the muscle, whereas the cross-connections in rigor muscle were angled at around 45° to the fibre axis, as if the crossbridges were ‘rowing’ actin past myosin. Hugh Huxley summarised these and other structural ideas about the crossbridge cycle in a classic paper in 1969 (Huxley, 1969[p]). The biochemical basis of the crossbridge cycle ATPase activity on actin was then put into a solid framework by the ground-breaking work of Lymn & Taylor (1971)[t], and the two put together as in Figure 14.

The results of Ramsey & Street (1940)[t] were interesting, but better measures of the variation of tension with sarcomere length were obtained by Andrew Huxley and his team at University College London, with the definitive results published in 1966 (Gordon et al., 1966[p]). They were able to define the sarcomere length quite accurately using a so-called ‘spot-follower’ system (monitoring the separation of two gold leaf markers attached to the fibre). If a relaxed fibre is stretched, the resting tension does not change a great deal until a sarcomere length is reached at which the tension starts to rise quite significantly. This is thought to be when the slack in the titin ‘spring’ has been taken up and titin starts to stretch.

**Figure 15.**
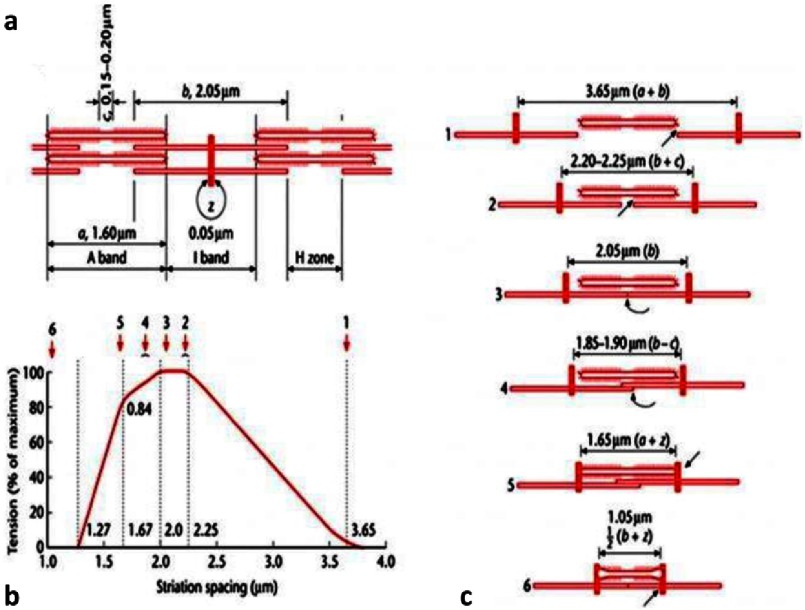
The active tension produced by the muscle at different sarcomere lengths (from Gordon et al., 1966[p]). If the myosin heads or crossbridges act as independent force generators, then, as the sarcomere length (S) is increased and the overlap of the actin and myosin filaments reduces (b), the tension produced by the muscle should gradually reduce in proportion to the overlap. A linear reduction in tension was observed as the sarcomere length changed from about 2.2 µm to about 3.6 µm (labelled as (1)). Since the actin filaments are about 1 µm long (a) and separated by an estimated Z-band thickness of 0.05 µm, and since the myosin filament length is about 1.6 µm, it would be expected that there would be zero overlap and hence zero tension when S is greater than or equal to 3.65 µm (=1.6 μm + 1.0 μm + 1.0 μm + 0.05 μm). As the sarcomere length is reduced the overlap will gradually increase until the two bridge regions of the myosin filaments are fully overlapped by actin. This will occur at a sarcomere length of about 2.25 µm (2 × 1.0 µm for the actin filaments plus 0.05 µm for the Z-band, plus the size of the bare zone of be about 0.2 µm, labelled as (2)). As summarised in (c), reduction of S below this value would not increase the number of interacting crossbridges any further so there will be an active tension plateau as observed between 2 and 3 µm. After this there are complications to the simple analysis; first the actin filaments meet the M-band, then there is overlap of anti-parallel actin filaments, the actin filaments then start overlapping myosin bridge regions with the wrong polarity in the other half of the A-band, and finally the myosin filaments bump up against the Z-bands, so the observed tension gradually reduces below S = 2.0 μm. Adapted from Gordon et al. (1966)[t] and reproduced with permission.

The active tension of fibres therefore needs to be calculated by subtracting the resting tension from the total tension measured at a given sarcomere length. When this was done by Gordon et al. (1966)[t], they found that the observed active tension (Figure 15(b)) fitted almost exactly to what might be expected by sliding filaments if the side-pieces or crossbridges act as independent force generators and tension is a function of the amount of overlap between the myosin and actin filaments. Figure 15(b) and (c) shows what happens at different sarcomere lengths, including tension staying level when the actin filaments cross the myosin filament bare zone (2,3), and tension dropping first when opposing arrays of actin filaments clashed at the M-band (4) and then when myosin filaments collided with the Z-line (5). There was little doubt after all of these studies that the mechanism of sliding filaments with independent force generators was here to stay.

## Do the myosin and actin filaments actually change length?

**Figure 16.**
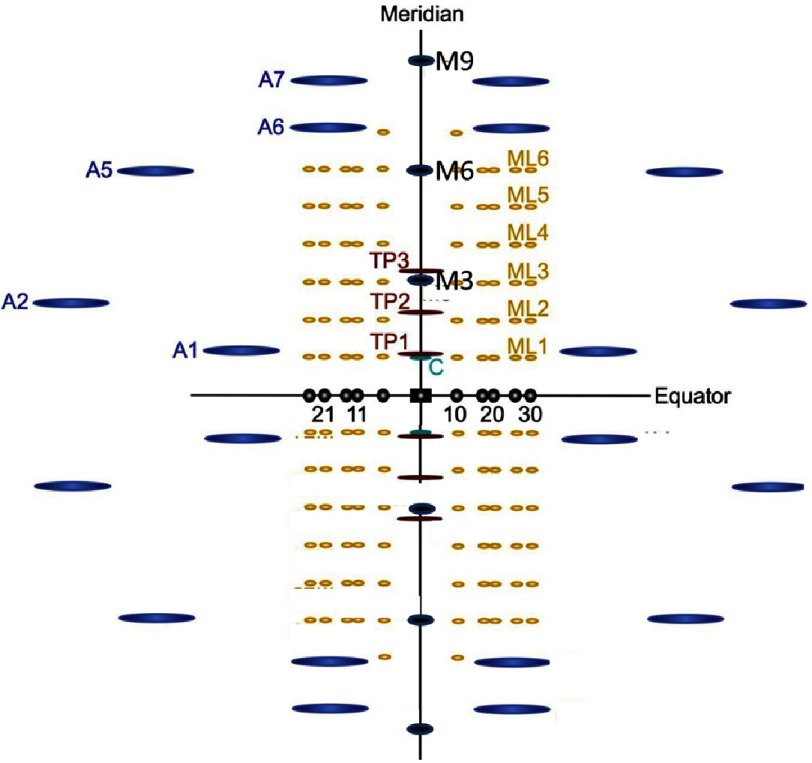
Schematic representation of an X-ray diffraction pattern from a fish muscle (Harford & Squire, 1986[p]). The right and left portions of the pattern are related by mirror symmetry about the meridian and the top and bottom by mirror symmetry across the equator. The equatorial reflections (marked with indices 10, 11, 20, 21, and 30; see the 10 and 11 in Figure 9(a)) are shown in black and are produced by radiation scattered from both the myosin and the actin filaments at right angles to the fibre axis. The yellow layer-line reflections are produced only by the myosin filaments and are labelled ML1 to ML6 (ML stands for Myosin Layer-line). They relate to successive orders of a ‘d’ spacing in Bragg’s Law of 43 nm. The layer lines in blue are produced by the actin filaments (orders of d ∼ 36 nm) and are labelled A1 to A7 (A stands for Actin reflections). The main meridional reflections, in green, named M3, M6 and M9 (M for Myosin; orders of d = 14.3 nm) tell us about the one dimensional projection of the density of the myosin heads onto the muscle fibre axis (heads/ crossbridges on myosin and, in active or rigor muscle, heads on actin too). Reflections on the meridian labelled TP1 to TP3 (orders of d = 38.5 nm), are from the regulatory protein troponin on the actin filaments (see Figure 2(a)) and are shown in red. Figure courtesy of Dr. Carlo Knupp.

Some of the early X-ray diffraction work on muscle by Hugh Huxley and his collaborators (e.g. Huxley et al., 1965[p]), and also by Gerald Elliott (no relation to Arthur Elliott) and his team (e.g. Elliott, 1964[p]), studied the axial diffraction pattern as a function of the muscle state (for details and more historical descriptions see Squire, 2013; Hitchcock-Degregori & Irving, 2014[p]). Both teams showed what are called layer lines and meridional reflections (Figure 16), both of which come from the axial structure of the myosin and actin filaments, and they were able to show that the axial positions of these X-ray reflections did not change within experimental error if a resting muscle was activated or put into rigor. This was another argument in favour of sliding filaments. However, all of these observations, together with all the light microscopy and electron microscopy studies described earlier, might suggest that actin and myosin filaments are perfectly rigid rods. But of course they are not; they are protein assemblies that undergo thermal vibrations and in fact they do display elasticity.

The first evidence that these filaments can stretch, even if only by a small amount, was obtained by measuring sarcomere component lengths in electron micrographs of stretched rigor fibres (Suzuki & Sugi, 1983[p]).

More definitive evidence was then obtained using X-ray diffraction by Wakabayashi et al. (1994)[t] and Huxley et al. (1994)[t]. These later two studies looked at some of the outer meridional reflections from myosin and actin filaments, and they found that when the muscle carried full isometric tension, these reflections moved a tiny amount (about 0.3 to 0.5%), showing that the filaments themselves were stretching slightly under tension, as one might expect.

Many early studies of muscle mechanics, especially studies trying to estimate the number of myosin crossbridges attached to actin at any one time, or the elastic properties of the crossbridges themselves, assumed that the myosin and actin filaments were rigid. The fact that they are compliant really complicates this calculation and much rethinking about crossbridge compliance and attachment number has gone on since these two classic papers in 1994. But that is another story.

## Conclusions

The work of four giants of the muscle field, Hugh Huxley, Andrew Huxley, Jean Hanson and Rolf Niedergerke established beyond reasonable doubt the sliding filament mechanism of muscle contraction, which applies to every kind of muscle that has been studied.

In fact so much was known about muscle, about myosin and actin filaments, about crossbridges, about how muscle was switched on and off (yet another story; see Squire, 2010[p]), and about the actomyosin ATPase cycle, that in 1972, when there was a Cold Spring Harbor Symposium devoted to muscle contraction (published as Cold Spring Harbor Symposia on Quantitative Biology, No. 37; 1973), there was a general feeling among the participants that very soon there would not be much more to learn about how muscle works. How wrong they were.

Now in 2016, when this article is being written, we still do not know (although there are lots of ideas): how many heads are attached to actin in a fully active muscle, what the crossbridge compliance is, exactly what the structural changes in the crossbridges are that are associated with force generation, what the biochemical states are that are associated with force production, what the details of the actin filament regulatory system are, how much regulation is carried out by myosin filaments and so on. There are also many enzymes and ancillary proteins in different muscles, apart from myosin, actin and titin, whose exact roles have yet to be determined.

For recent papers or reviews of current ideas on filament structures and the contractile mechanism see, for example, Squire et al. (2005)[t]; Geeves & Holmes (2005)[t]; Sweeney & Houdusse (2010)[t]; Behrmann et al. (2012)[t]; Al-Khayat et al. (2013)[t]. In short, there is still plenty for muscle researchers to do, especially since many of these proteins can carry mutations which are associated with various myopathies of skeletal and cardiac muscles. Much of the emphasis of muscle research at the moment is to try to understand the origin of various cardiomyopathies. The sliding filament model for muscle is almost like setting the stage. Now we need to know what the various actors actually do to get the whole muscle show on the road, and what happens when the players forget their lines.
